# Extensor Pollicis Longus Tendon Rupture Following a Cat Bite: A Case Report and Review of Literature

**DOI:** 10.7759/cureus.43940

**Published:** 2023-08-22

**Authors:** Sushmit Singh, Salim Adamji, Ravi Badge

**Affiliations:** 1 Trauma and Orthopaedics, Warrington and Halton Hospitals NHS Foundation Trust, Warrington, GBR

**Keywords:** tendon surgery, hand injuries, rare case report, cat-bite, extensor tendon rupture

## Abstract

This case report describes the first reported occurrence of extensor pollicis longus (EPL) tendon rupture caused by a cat bite. Animal bites, particularly from cats, can cause various complications, including damage to tendons and bones. In our case, a 43-year-old female suffered an EPL rupture in her dominant hand after being bitten by her cat. The patient underwent EPL reconstruction by means of extensor indicis proprius (EIP) using standard techniques, with a positive functional outcome reported. The case highlights the importance of considering the risk of tendon injuries following animal bites and prompt treatment to prevent permanent disability. This report calls for a high index of suspicion for tendon injury following animal bites, even if there are no signs of infection.

## Introduction

Animal bites frequently lead to hand injuries, with cat bites being the second most common cause, accounting for approximately 5% of such incidents [1,2]. The sharp teeth of cats are capable of penetrating deep into tissues and can cause a range of complications, including infection, soft tissue injuries, and damage to tendons and bones [2]. In this case report, we present the first reported case of extensor pollicis longus (EPL) tendon rupture following a cat bite. While there are established complications associated with distal radius fractures and extensor tendon injuries due to fight bites, to our knowledge, there are no reports of EPL tendon rupture following animal bites. This case highlights the importance of considering the risk of tendon injuries following animal bites and the need for prompt and appropriate treatment to prevent permanent disability.

## Case presentation

**Figure 1:**
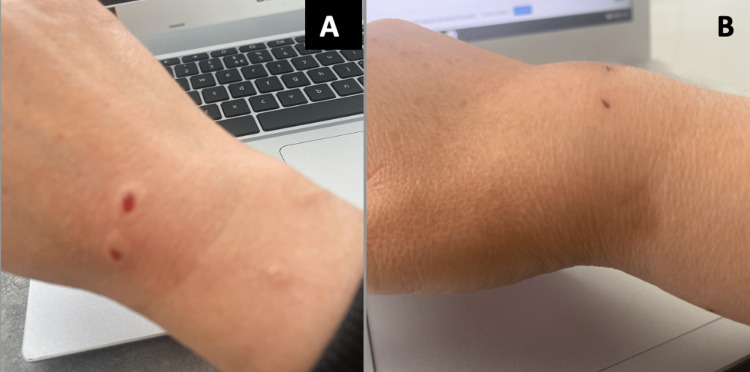
A&B: Pre-operative pictures of the cat bite mark

A 43-year-old woman visited her general practitioner nine days after suffering bite injuries to her right hand from her cat. She experienced an inability to straighten her thumb, which progressively worsened since the injury and eventually could not straighten without manual assistance. Blood tests and X-rays yielded no abnormalities. Subsequently, she was referred to an orthopaedic outpatient clinic. At the nine-week mark post-injury, an ultrasound scan revealed a torn and retracted EPL. Unfortunately, due to appointment cancellations, she was seen almost three months after the injury. On history and examination, other risk factors, such as rheumatoid arthritis, gout, fractures and deformities, were meticulously excluded. On clinical examination, two bite marks were evident on the dorsum of her wrist, aligned with the path of the EPL (Figure 1 A&B). These wounds were non-painful upon examination. Notably, she exhibited restricted extension at the interphalangeal joint and insufficient thumb retropulsion. No signs of localized infection or distal neurovascular impairment were noted during the examination. After a thorough discussion regarding treatment options, the patient chose to proceed with EPL reconstruction using the ipsilateral functional EIP tendon.

**Figure 2:**
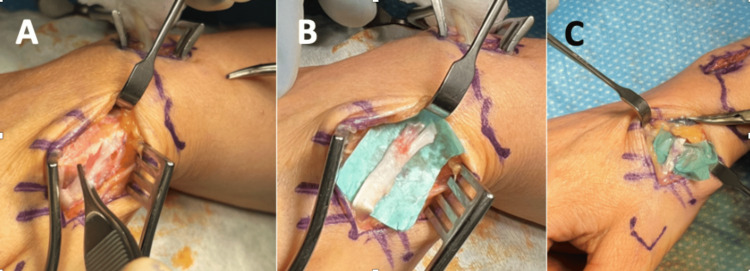
Intra-operative pictures showing: A&B, Completely torn EPL tendon; C, EIP to EPL transfer performed and secured with sutures EIP: extensor indicis proprius; EPL: extensor indicis proprius

The patient underwent a procedure for reconstruction of the right thumb EPL after suffering a tendon rupture caused by a previous cat bite. The intervention encompassed a three-incision approach [3]. First, an incision proximal to the index metacarpophalangeal joint was made, enabling the liberation of the EIP tendon up to the level of the wrist retinaculum. Second, a distal incision beyond the retinaculum was performed, allowing retrieval of the EIP tendon, followed by a third incision directly over the metacarpal-level EPL tendon to access its distal stump. Notably, there were no indications of infection during the surgical procedure. Removal of irregular scar tissue was carried out, which had developed around the previous cat bite marks. The degenerated ends of the torn EPL tendon were excised, and then freshened (Figure 2 A&B). Subsequently, the EIP tendon was passed subcutaneously and anchored using 4.0 PDS in a Pulvertaft weave configuration (Figure 2 C) [4]. The efficacy of the tenodesis effect was verified by passive wrist movement from flexion to extension, revealing that finger positioning exhibited a symmetrical cascade, thereby confirming satisfactory fixation.

Following the procedure, the patient's wrist was placed in a back slab with 20-degree dorsiflexion and the thumb was in a neutral position for two weeks. The rehabilitation protocol was done in a phased approach with initial weeks (0-3) focused on splint usage and gentle thumb exercises. Weeks 3-5 integrated splint wear, thumb abduction exercises, and scar management. Weeks 5-6 emphasized controlled strengthening and employing a splint during activities. Beyond week 6, the splint was worn at night and during light tasks such as driving. The patient gradually engaged in strengthening exercises, returned to manual activities, and sought therapist guidance for more demanding tasks.

On the final follow-up after three months of the surgery, the patient demonstrated improvement in the active retropulsion and extension of her thumb, indicating a positive functional outcome. The patient was happy with the outcome, as she was managing well with all her activities.

## Discussion

This case report highlights an unusual and rare complication of cat bites that is not commonly encountered in clinical practice, namely, an extensor pollicis longus (EPL) tendon rupture. Although EPL ruptures can result from various causes, it is extremely rare to see them associated with a cat bite [1]. In this case, the mechanism of injury is thought to be the bite-induced trauma to the tendon, which led to partial rupture, followed by complete rupture over time.

While the current case is the first reported case in English literature highlighting this unusual complication following a cat bite, it is important to note that EPL ruptures can occur due to various causes. The review of existing literature on extensor pollicis tendon ruptures revealed only a handful of cases caused by animal bites, with cat bites being relatively uncommon. These include tendon ruptures due to surgical management [5], direct trauma, rheumatoid arthritis [6], infiltration of tophaceous gout [7], ankylosing spondylitis, the development of bone spurs following metastatic distal radius or scaphoid fractures [8], or Madelung’s deformity [9].

The injury could potentially result from the force of the bite, combined with the tendon's location and vulnerability to damage. The presented case highlights the possibility of EPL rupture due to a bite wound over the dorsal aspect of the wrist and emphasizes the need to have a high index of suspicion for tendon injury following animal bites. The case also underscores the importance of a thorough evaluation and prompt management of patients with animal bites to prevent complications.

It is important to note that the risk of infection, possibly through Pasteurella multocida [10], should also be considered when managing this condition since cat bites can lead to infections that could potentially exacerbate or complicate the rupture. It is crucial to evaluate the wound thoroughly for possible infection and provide appropriate treatment, including tetanus immunization, wound cleansing, and administration of antibiotics.

## Conclusions

In conclusion, this case report describes a rare and unusual case of EPL tendon rupture caused by a cat bite. Healthcare providers should be aware of the potential for EPL tendon rupture in the context of animal bites and should consider this diagnosis in patients presenting with symptoms such as swelling and limited range of motion following a bite wound. Appropriate management, including thorough evaluation for possible infection and prompt surgical repair of the affected tendon, is critical to achieving a successful outcome.
